# 2-Chloro­benzene­sulfonamide

**DOI:** 10.1107/S1600536809031511

**Published:** 2009-08-15

**Authors:** B. Thimme Gowda, Sabine Foro, K. Shakuntala, Hartmut Fuess

**Affiliations:** a Department of Chemistry, Mangalore University, Mangalagangotri 574 199, Mangalore, India; b Institute of Materials Science, Darmstadt University of Technology, Petersenstrasse 23, D-64287 Darmstadt, Germany

## Abstract

In the crystal of the title compound, C_6_H_6_ClNO_2_S, N—H⋯O hydrogen bonds pack the mol­ecules into sheets parallel to the *ac* plane.

## Related literature

For our studies of the effect of substituents on the solid state structures of sulfonamides and *N*-halo aryl­sulfonamides, see: Gowda *et al.* (2003 ▶); Gowda, Babitha *et al.* (2007 ▶); Gowda, Nayak *et al.* (2007 ▶); Gowda, Srilatha *et al.* (2007 ▶). For the parent benzene­sulfonamide, see: Gowda, Nayak *et al.* (2007 ▶). For other aryl sulfonamides, see: Gowda, Babitha *et al.* (2007 ▶); Gowda, Srilatha *et al.* (2007 ▶); Jones & Weinkauf (1993 ▶); Kumar *et al.* (1992 ▶); O’Connor & Maslen (1965 ▶).
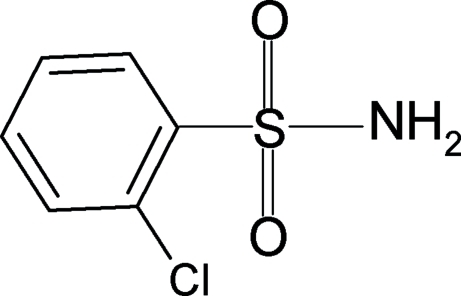


## Experimental

### 

#### Crystal data

C_6_H_6_ClNO_2_S
*M* *_r_* = 191.63
Monoclinic, 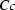

*a* = 6.955 (1) Å
*b* = 14.848 (3) Å
*c* = 7.751 (1) Å
β = 91.51 (1)°
*V* = 800.2 (2) Å^3^
*Z* = 4
Mo *K*α radiation
μ = 0.68 mm^−1^
*T* = 299 K
0.48 × 0.48 × 0.26 mm

#### Data collection

Oxford Diffraction Xcalibur diffractometer with a Sapphire CCD detector
Absorption correction: multi-scan (*CrysAlis RED*; Oxford Diffraction, 2009 ▶) *T*_min_ = 0.735, *T*_max_ = 0.842
1598 measured reflections
1031 independent reflections
1004 reflections with *I* > 2σ(*I*)
*R*_int_ = 0.008

#### Refinement

*R*[*F* ^2^ > 2σ(*F* ^2^)] = 0.022
*wR*(*F* ^2^) = 0.060
*S* = 1.03
1031 reflections
106 parameters
4 restraints
H atoms treated by a mixture of independent and constrained refinement
Δρ_max_ = 0.14 e Å^−3^
Δρ_min_ = −0.21 e Å^−3^
Absolute structure: Flack (1983 ▶), 215 Friedel pairs
Flack parameter: 0.04 (8)

### 

Data collection: *CrysAlis CCD* (Oxford Diffraction, 2009 ▶); cell refinement: *CrysAlis RED* (Oxford Diffraction, 2009 ▶); data reduction: *CrysAlis RED*; program(s) used to solve structure: *SHELXS97* (Sheldrick, 2008 ▶); program(s) used to refine structure: *SHELXL97* (Sheldrick, 2008 ▶); molecular graphics: *PLATON* (Spek, 2009 ▶); software used to prepare material for publication: *SHELXL97*.

## Supplementary Material

Crystal structure: contains datablocks I, global. DOI: 10.1107/S1600536809031511/bt5028sup1.cif

Structure factors: contains datablocks I. DOI: 10.1107/S1600536809031511/bt5028Isup2.hkl

Additional supplementary materials:  crystallographic information; 3D view; checkCIF report

## Figures and Tables

**Table 1:** Hydrogen-bond geometry (Å, °)

*D*—H⋯*A*	*D*—H	H⋯*A*	*D*⋯*A*	*D*—H⋯*A*
N1—H11⋯O1^i^	0.857 (19)	2.12 (2)	2.908 (3)	152 (3)
N1—H12⋯O2^ii^	0.835 (18)	2.12 (2)	2.941 (3)	166 (3)

Symmetry codes: (i) 
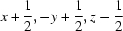
; (ii) 
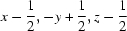
.

**Fig. 1.**
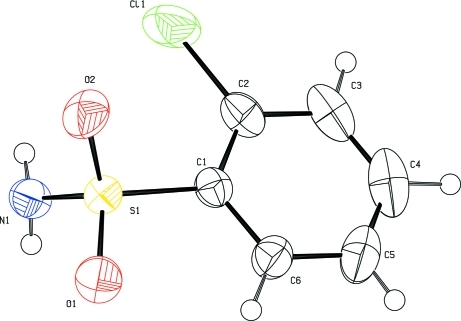
Molecular structure of (I), showing the atom labelling scheme and displacement ellipsoids are drawn at the 50% probability level.

**Fig. 2.**
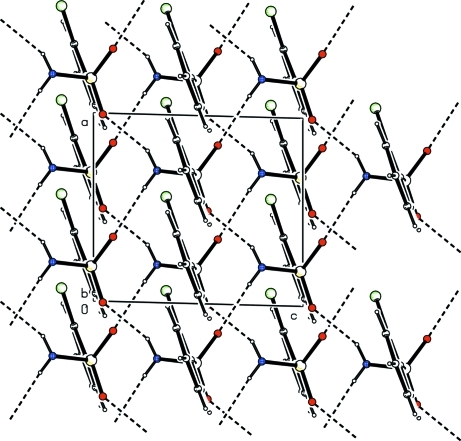
Molecular packing of (I) with hydrogen bonding shown as dashed lines.

## supplementary crystallographic information

### Comment

The chemistry of sulfonamides is of interest as they show distinct physical, chemical and biological properties. Many arylsulfonamides and their N-halo compounds exhibit pharmacological, fungicidal and herbicidal activities due to their oxidizing action in aqueous, partial aqueous and non-aqueous media. In the present work, the structure of 2-chlorobenzenesulfonamde  has been determined to explore the substituent effects on the solid state structures of sulfonamides and N-halo arylsulfonamides (Gowda *et al.*, 2003; Gowda, Babitha *et al.*  2007; Gowda, Nayak *et al.*  2007; Gowda, Srilatha *et al.*  2007). The structure of  the title compound (Fig. 1) closely resembles those of the parent benzenesulfonamide (Gowda, Nayak *et al.*, 2007) and other aryl sulfonamides (Gowda, Babitha *et al.*, 2007; Gowda, Srilatha *et al.*, 2007; Jones & Weinkauf, 1993; Kumar *et al.*, 1992; O'Connor & Maslen, 1965). The title compound crystallizes in monoclinic space group Cc in contrast to the monoclinic *Pc* space group observed with the parent sulfonamide (Gowda *et al.*, 2007*b*) and orthorhombic Pbca space group with 4-fluorobenzenesulfonamide (Jones & Weinkauf, 1993) and 4-aminobenzenesulfonamide (O'Connor & Maslen, 1965) and monoclinic P2_1_/n space group with 4-chlorobenzenesulfonamide and 4-bromobenzenesulfonamide (Gowda *et al.*, 2003), and 4-methylbenzenesulfonamide (Kumar *et al.*, 1992). The molecules in the title compound are packed into infinite 3-D molecular network through N1—H11···O1 and N1—H12···O2 hydrogen bonding (Table 1 & Fig.2).

### Experimental

The purity of the commmercial sample (TCI, Tokyo) was checked and characterized by its infrared spectra. The single crystals used in X-ray diffraction studies were grown in ethanol solution by a slow evaporation of the solvent at room temperature.

### Refinement

The H atoms of the NH_2_ group were located in difference map and refined with restrained geometry to 0.86 (2) Å. The other H atoms were positioned with idealized geometry using a riding model [C—H = 0.93 Å]. All H atoms were refined with isotropic displacement parameters (set to 1.2 times of the *U*_eq_ of the parent atom).

### Crystal data

**Table:**

C_6_H_6_ClNO_2_S	*F*(000) = 392
*M**_r_* = 191.63	*D*_x_ = 1.591 Mg m^−^^3^
Monoclinic, *C**c*	Mo *K*α radiation, λ = 0.71073 Å
Hall symbol: C -2yc	Cell parameters from 1188 reflections
*a* = 6.955 (1) Å	θ = 2.6–27.8°
*b* = 14.848 (3) Å	µ = 0.68 mm^−^^1^
*c* = 7.751 (1) Å	*T* = 299 K
β = 91.51 (1)°	Prism, colourless
*V* = 800.2 (2) Å^3^	0.48 × 0.48 × 0.26 mm
*Z* = 4	

### Data collection

**Table:**

Oxford Diffraction Xcalibur diffractometer with a Sapphire CCD detector	1031 independent reflections
Radiation source: fine-focus sealed tube	1004 reflections with *I* > 2σ(*I*)
graphite	*R*_int_ = 0.008
Rotation method data acquisition using ω and phi scans.	θ_max_ = 26.4°, θ_min_ = 2.7°
Absorption correction: multi-scan (*CrysAlis RED*; Oxford Diffraction, 2009)	*h* = −8→4
*T*_min_ = 0.735, *T*_max_ = 0.842	*k* = −18→15
1598 measured reflections	*l* = −9→9

### Refinement

**Table:**

Refinement on *F*^2^	Secondary atom site location: difference Fourier map
Least-squares matrix: full	Hydrogen site location: inferred from neighbouring sites
*R*[*F*^2^ > 2σ(*F*^2^)] = 0.022	H atoms treated by a mixture of independent and constrained refinement
*wR*(*F*^2^) = 0.060	*w* = 1/[σ^2^(*F*_o_^2^) + (0.0417*P*)^2^ + 0.2306*P*] where *P* = (*F*_o_^2^ + 2*F*_c_^2^)/3
*S* = 1.03	(Δ/σ)_max_ = 0.041
1031 reflections	Δρ_max_ = 0.14 e Å^−^^3^
106 parameters	Δρ_min_ = −0.21 e Å^−^^3^
4 restraints	Absolute structure: Flack (1983), 215 Friedel pairs
Primary atom site location: structure-invariant direct methods	Flack parameter: 0.04 (8)

### Special details

**Table:**

Experimental. CrysAlis RED (Oxford Diffraction, 2009) Empirical absorption correction using spherical harmonics, implemented in SCALE3 ABSPACK scaling algorithm.
Geometry. All e.s.d.'s (except the e.s.d. in the dihedral angle between two l.s. planes) are estimated using the full covariance matrix. The cell e.s.d.'s are taken into account individually in the estimation of e.s.d.'s in distances, angles and torsion angles; correlations between e.s.d.'s in cell parameters are only used when they are defined by crystal symmetry. An approximate (isotropic) treatment of cell e.s.d.'s is used for estimating e.s.d.'s involving l.s. planes.
Refinement. Refinement of *F*^2^ against ALL reflections. The weighted *R*-factor *wR* and goodness of fit *S* are based on *F*^2^, conventional *R*-factors *R* are based on *F*, with *F* set to zero for negative *F*^2^. The threshold expression of *F*^2^ > σ(*F*^2^) is used only for calculating *R*-factors(gt) *etc*. and is not relevant to the choice of reflections for refinement. *R*-factors based on *F*^2^ are statistically about twice as large as those based on *F*, and *R*- factors based on ALL data will be even larger.

### Fractional atomic coordinates and isotropic or equivalent isotropic displacement parameters (Å^2^)

**Table:**

	*x*	*y*	*z*	*U*_iso_*/*U*_eq_	
Cl1	0.56409 (10)	0.10747 (7)	0.84733 (11)	0.0700 (3)	
S1	0.18379 (8)	0.20974 (3)	0.98884 (8)	0.03276 (14)	
O1	−0.0067 (3)	0.22742 (12)	1.0448 (3)	0.0486 (5)	
O2	0.3434 (3)	0.23916 (13)	1.0939 (2)	0.0535 (5)	
N1	0.2010 (4)	0.25747 (15)	0.8048 (3)	0.0396 (5)	
H11	0.309 (3)	0.251 (2)	0.755 (4)	0.048*	
H12	0.105 (4)	0.249 (2)	0.740 (3)	0.048*	
C1	0.1995 (4)	0.09052 (15)	0.9649 (3)	0.0335 (5)	
C2	0.3626 (4)	0.04740 (18)	0.9082 (4)	0.0443 (6)	
C3	0.3663 (6)	−0.0459 (2)	0.8966 (4)	0.0588 (8)	
H3	0.4755	−0.0751	0.8582	0.071*	
C4	0.2078 (7)	−0.0951 (2)	0.9421 (4)	0.0674 (10)	
H4	0.2106	−0.1576	0.9341	0.081*	
C5	0.0464 (6)	−0.05329 (19)	0.9990 (4)	0.0590 (8)	
H5	−0.0600	−0.0872	1.0292	0.071*	
C6	0.0415 (4)	0.04025 (18)	1.0117 (4)	0.0432 (6)	
H6	−0.0677	0.0689	1.0515	0.052*	

### Atomic displacement parameters (Å^2^)

**Table:**

	*U*^11^	*U*^22^	*U*^33^	*U*^12^	*U*^13^	*U*^23^
Cl1	0.0380 (4)	0.0856 (6)	0.0871 (6)	0.0153 (4)	0.0158 (4)	0.0148 (5)
S1	0.0338 (3)	0.0335 (2)	0.0310 (2)	−0.0015 (3)	0.00213 (18)	−0.0023 (2)
O1	0.0468 (11)	0.0480 (9)	0.0520 (11)	0.0068 (9)	0.0205 (9)	−0.0043 (9)
O2	0.0590 (14)	0.0501 (11)	0.0506 (11)	−0.0152 (10)	−0.0172 (10)	−0.0031 (9)
N1	0.0364 (11)	0.0449 (11)	0.0375 (11)	0.0035 (10)	0.0025 (8)	0.0055 (9)
C1	0.0381 (12)	0.0343 (10)	0.0280 (11)	0.0023 (11)	−0.0025 (9)	0.0013 (8)
C2	0.0478 (16)	0.0476 (13)	0.0374 (13)	0.0118 (13)	−0.0008 (12)	0.0047 (11)
C3	0.078 (2)	0.0498 (15)	0.0485 (15)	0.0276 (17)	−0.0026 (15)	0.0016 (13)
C4	0.116 (3)	0.0360 (14)	0.0497 (18)	0.0069 (19)	−0.0055 (19)	0.0025 (12)
C5	0.079 (2)	0.0399 (14)	0.0574 (18)	−0.0158 (15)	−0.0058 (17)	0.0052 (13)
C6	0.0445 (16)	0.0412 (12)	0.0436 (14)	−0.0049 (12)	−0.0026 (12)	0.0022 (11)

### Geometric parameters (Å, °)

**Table:**

Cl1—C2	1.737 (3)	C2—C3	1.389 (4)
S1—O1	1.429 (2)	C3—C4	1.376 (5)
S1—O2	1.4275 (19)	C3—H3	0.9300
S1—N1	1.600 (2)	C4—C5	1.366 (5)
S1—C1	1.784 (2)	C4—H4	0.9300
N1—H11	0.857 (19)	C5—C6	1.393 (4)
N1—H12	0.835 (18)	C5—H5	0.9300
C1—C6	1.385 (4)	C6—H6	0.9300
C1—C2	1.384 (4)		
			
O1—S1—O2	118.92 (13)	C3—C2—Cl1	118.6 (2)
O1—S1—N1	106.32 (13)	C4—C3—C2	119.8 (3)
O2—S1—N1	107.32 (12)	C4—C3—H3	120.1
O1—S1—C1	105.88 (12)	C2—C3—H3	120.1
O2—S1—C1	108.31 (12)	C5—C4—C3	120.8 (3)
N1—S1—C1	109.91 (11)	C5—C4—H4	119.6
S1—N1—H11	116 (2)	C3—C4—H4	119.6
S1—N1—H12	113 (2)	C4—C5—C6	119.8 (3)
H11—N1—H12	114 (3)	C4—C5—H5	120.1
C6—C1—C2	119.8 (2)	C6—C5—H5	120.1
C6—C1—S1	117.2 (2)	C1—C6—C5	119.9 (3)
C2—C1—S1	123.0 (2)	C1—C6—H6	120.1
C1—C2—C3	119.9 (3)	C5—C6—H6	120.1
C1—C2—Cl1	121.5 (2)		
			
O1—S1—C1—C6	−3.7 (2)	S1—C1—C2—Cl1	−2.5 (3)
O2—S1—C1—C6	124.8 (2)	C1—C2—C3—C4	−0.2 (4)
N1—S1—C1—C6	−118.19 (19)	Cl1—C2—C3—C4	−179.3 (2)
O1—S1—C1—C2	178.5 (2)	C2—C3—C4—C5	−0.1 (4)
O2—S1—C1—C2	−52.9 (2)	C3—C4—C5—C6	−0.1 (5)
N1—S1—C1—C2	64.0 (2)	C2—C1—C6—C5	−1.0 (4)
C6—C1—C2—C3	0.7 (4)	S1—C1—C6—C5	−178.8 (2)
S1—C1—C2—C3	178.4 (2)	C4—C5—C6—C1	0.7 (4)
C6—C1—C2—Cl1	179.77 (19)		

### Hydrogen-bond geometry (Å, °)

**Table:**

*D*—H···*A*	*D*—H	H···*A*	*D*···*A*	*D*—H···*A*
N1—H11···O1^i^	0.86 (2)	2.12 (2)	2.908 (3)	152 (3)
N1—H12···O2^ii^	0.84 (2)	2.12 (2)	2.941 (3)	166 (3)

Symmetry codes: (i) *x*+1/2, −*y*+1/2, *z*−1/2; (ii) *x*−1/2, −*y*+1/2, *z*−1/2.

## References

[bb1] Flack, H. D. (1983). *Acta Cryst.* A**39**, 876–881.

[bb2] Gowda, B. T., Babitha, K. S., Svoboda, I. & Fuess, H. (2007). *Acta Cryst.* E**63**, o3245. 10.1107/S1600536807062137 PMC2915044 21200965

[bb3] Gowda, B. T., Jyothi, K., Kozisek, J. & Fuess, H. (2003). *Z. Naturforsch. Teil A*, **58**, 656–660.

[bb4] Gowda, B. T., Nayak, R., Kožíšek, J., Tokarčík, M. & Fuess, H. (2007). *Acta Cryst.* E**63**, o2967.

[bb5] Gowda, B. T., Srilatha, Foro, S., Kozisek, J. & Fuess, H. (2007). *Z. Naturforsch. Teil A*, **62**, 417–424.

[bb6] Jones, P. G. & Weinkauf, A. (1993). *Z. Kristallogr.* **208**, 128–129.

[bb7] Kumar, S. V., Senadhi, S. E. & Rao, L. M. (1992). *Z. Kristallogr.* **202**, 1–6.

[bb8] O’Connor, B. H. & Maslen, E. N. (1965). *Acta Cryst.* **18**, 363–366.

[bb9] Oxford Diffraction (2009). *CrysAlis CCD* and *CrysAlis RED* Oxford Diffraction Ltd, Yarnton, England.

[bb10] Sheldrick, G. M. (2008). *Acta Cryst.* A**64**, 112–122. 10.1107/S0108767307043930 18156677

[bb11] Spek, A. L. (2009). *Acta Cryst.* D**65**, 148–155. 10.1107/S090744490804362X PMC2631630 19171970

